# Mechanistic study of quercetin in the treatment of thyroid cancer with diabetes based on network pharmacology and *in vitro* experiments

**DOI:** 10.3389/fendo.2025.1537799

**Published:** 2025-06-12

**Authors:** Siyu Liu, Yujie Cai, Wenjing Chen, Xiao Yuan, Zhipeng He, Feng Lin

**Affiliations:** ^1^ Department of General Surgery, The First Affiliated Hospital of Anhui Medical University, Hefei, China; ^2^ Anhui Public Health Clinical Center, Hefei, China; ^3^ Department of Radiology, Zhejiang Cancer Hospital, Hangzhou Institute of Medicine (HIM), Chinese Academy of Sciences, Hangzhou, China; ^4^ School of Pharmacy, Jiangxi Medical College, Nanchang University, Nanchang, China; ^5^ Department of General Surgery, The First Affiliated Hospital of Zhejiang Chinese Medical University (Zhejiang Provincial Hospital of Chinese Medicine), Hangzhou, China

**Keywords:** quercetin, thyroid cancer, diabetes, network pharmacology, bioinformatics, molecular docking

## Abstract

**Introduction:**

Thyroid cancer (TC) stands as a prevalent malignancy within the global endocrine system, with its incidence notably exacerbated by the presence of diabetes. However, the specific relationship between TC and diabetes and promising treatment strategies that address both conditions simultaneously are still under exploration. Quercetin, herb medicine from traditional Chinese medicine (TCM), is widely used as an adjunctive therapy with Western medicine in the treatment of many diseases based on a wide range of biological effects. The objective of this study was to explore the efficacy of quercetin in treating TC with diabetes combining bioinformatics and network pharmacology.

**Methods:**

After multistep Cox proportional hazards regressions, we built a prognostic risk model for TC-diabetes and identified targets for quercetin in treating TC with diabetes. Molecular docking was employed to evaluate the binding affinities of quercetin with core targets, and *in vitro* experiments verified quercetin’s targets and functions.

**Results:**

11 prognostic genes were included in the prediction model with a great performance in predicting the prognosis of TC-diabetic patients. 45 genes served as the targets of quercetin in treating TC with diabetes, among which, 5 core genes were screened as the most contributors. Through molecular docking, matrix metalloproteinase-3 (MMP3) was identified as the potential therapeutic target of quercetin. In vitro experiments have found that quercetin can inhibit the proliferation of thyroid cancer cells and the expression of MMP3 under high glucose conditions.

**Discussion:**

In summary, quercetin may suppress the progression of TC-diabetes by inhibiting the proliferation of thyroid cancer cells and the expression of MMP3.

## 1 Introduction

Thyroid cancer (TC) is the common malignant tumor in the endocrine system among populations, with a significant increase in prevalence worldwide over the past several decades, especially due predominantly to an increase in papillary thyroid cancer (PTC) (1, 2). The etiology of TC is multifactorial, including genetic, environmental, and social-psychological factors. Currently, the management of TC in clinical settings is promising with a relatively high five-year survival as surgical operation is an effective therapeutic approach for TC patients (1). Another common disease of the endocrine system is diabetes mellitus (DM), with typical features of hyperglycemia, hyperinsulinemia, and insulin resistance (3). Empirical evidence has indicated that DM acts as a risk factor that would lead to high prevalence and poor prognosis of TC (4). A previous review demonstrated that diabetes patients showed a marginally significant increased risk of developing thyroid cancer compared with those without diabetes (5). Moreover, in a case-control study, Bezin et al. found that the use of glucagon-like peptide 1 (GLP-1) receptor agonists in Type 2 DM patients could increase the risk of TC, especially after 1-3 years of treatment (6). However, the discovery that diabetes could increase the risk of TC remains controversial. Wang et al. revealed that diabetes and longer diabetes duration (> 5 years) showed no significant association with TC (7). Therefore, exploring the specific relationship between TC and diabetes and developing treatment strategies that address both conditions simultaneously have significant clinical importance.

While thyroid surgery, radioactive iodine therapy, and thyroid stimulating hormone suppression are the main approaches to TC treatment, they can be a double-edged sword accompanied by endocrine disorders and other complications (8). Currently, traditional Chinese medicine (TCM) has become an area of active interest owing to its flexible functions (9–12). Herb medicine from TCM is widely used as an adjunctive therapy with Western medicine in clinical settings (13). Quercetin is among the widely prevalent polyphenols, belonging to the flavonoid subclass of flavonols, and is found abundantly in fruits and vegetables (14, 15). The natural flavonoid of quercetin is beneficial to multi-system diseases by the modulation of various targets and signaling pathways based on a wide range of biological effects (16), such as anti-cancer, antioxidant, anti-diabetic, anti-inflammatory and angiogenesis inhibition functions (17, 18). For example, by regulating the Phosphoinositide 3-Kinase (PI3K)/Protein Kinase B (Akt)/Mammalian Target of Rapamycin (mTOR), Wnt/-catenin, and Mitogen-Activated Protein Kinase (MAPK)/Extracellular Signal-Regulated Kinase 1/2 (ERK1/2) pathways, quercetin could promote the loss of cell viability, apoptosis and autophagy and then exert anti-tumor effect (15). Strong evidence has demonstrated that quercetin is able to inhibit various types of cancers including breast, lung, nasopharyngeal, liver, kidney, colorectal, and ovarian cancer (19). In particular, quercetin can inhibit the growth, adhesion, and migration of thyroid cancer cells, and has redifferentiation properties in some thyroid cancer cell lines (20). Moreover, increasing research indicates that quercetin contributes to diabetes management (21) through the modulation of absorbing glucose, inhibiting the digestion of intestinal carbohydrates, promoting islet β cell regeneration, and reducing blood glucose levels (21, 22). It can also restrict insulin release in response to glucose via enhancing the extracellular regulated kinase 1/2 (23, 24). Notably, a recent study combining *in vitro* experiments suggests that quercetin can inhibit the progression of hepatocellular carcinoma with diabetes by regulating the Mitogen-activated protein kinase kinase (MEK)/ERK signaling pathway and prohibiting the proliferation and Epithelial-Mesenchymal Transition (EMT) of HepG2 cells (25). However, the potential molecular mechanisms underlying quercetin in treating TC with diabetes remain unknown and require further investigation.

**Figure 1:**
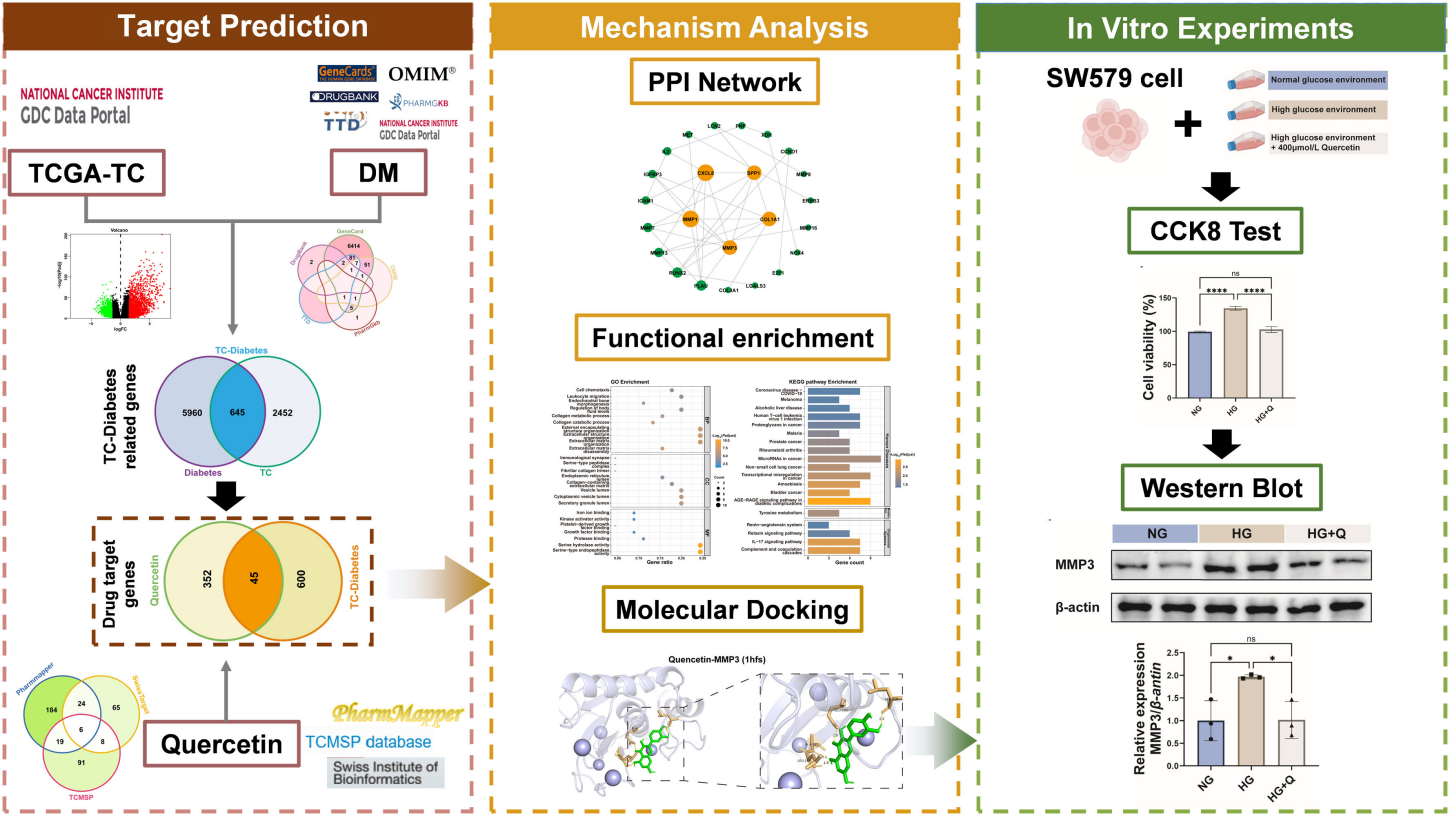
Flow chart. The target and mechanism of quercetin in the treatment of TC with diabetes. TC, Thyroid cancer; TC-Diabetes genes, Thyroid cancer with diabetes.

In the current study, we first evaluated the relationships between TC prognosis and DM by screening the targets of TC-diabetes and building a clinical prognostic risk model for TC-diabetes. Second, we identified the targets of quercetin in treating TC-diabetes and examined their biological functions through functional enrichment analyses. Third, molecular docking was employed to estimate the binding affinities of quercetin and core proteins related to TC-diabetes. Finally, we validated the anticancer effect of quercetin *in vitro* experiments through the inhibition of tumor metastasis (**Figure 1**). To conclude, this study systematically clarified the molecular mechanism by which quercetin inhibits the progression of thyroid cancer with DM through regulating key targets by constructing a clinical prognosis model of TC-Diabetes, specific targets screening and *in vitro* verification, providing new candidate targets and translational medical evidence for the precise treatment of TC-diabetes.

## 2 Methods

### 2.1 Identification and clinical analysis of TC−diabetes targets

The Cancer Genome Atlas (TCGA) database was analyzed to identify TC-related genes. Using the “DESeq” package in R language Bioconductor, we compared the gene expression matrices between tumor samples and normal samples to obtain the differentially expressed genes in TC. The significance level was set at FDR < 0.05 and |log^fold change(FC)^| > 1.5. The targets of diabetes were integrated from GeneCard, Online Mendelian Inheritance in Man (OMIM), DrugBank (26), PharmGkb (27), and Therapeutic Target Database (TTD) (28) databases and censored the repeated genes. These websites were user-friendly to provide available genetic information on human and diseases. Then, the TC-diabetes targets were defined as the overlapping genes between TC and diabetes targets. Using the “survival” package in R software, we constructed a prognostic risk model based on the univariate Cox regression, followed by lasso regression and multivariable Cox regression to evaluate the overall survival in TC-diabetes patients.

### 2.2 Screening of quercetin targets and therapeutic targets

The targets of quercetin were directly searched from the TCM Systems Pharmacology Database (TCMSP) (29), Swiss target prediction (30), and Pharmmapper databases as quercetin works as active compound of many herbs. We then limited the organism to “Homo sapiens” via the Universal Protein Resource (UniProt) database and removed the duplicated genes. The therapeutic targets of quercetin in TC-diabetes were then defined as the intersection between quercetin targets and TC-diabetes targets.

### 2.3 Protein–protein interaction and functional enrichment analyses

The therapeutic targets of quercetin in TC-diabetes were imported to the String database (31) to construct the protein–protein interaction (PPI) network and obtain the tab-separated values (TSV) data. The organism was set to “Homo sapiens”, the minimum interaction threshold was set to “high confidence” (> 0.7), and the rest were set as the default. Then, the TSV data was imported into Cytoscape 3.10.1 software (39) to build a PPI network. Finally, core genes were screened with CytoNCA plug-in by analyzing betweenness, closeness, degree, eigenvector, LAC, and network scores of the network. Among them, genes with larger degree values (>6) were regarded as the core ones. To evaluate the biological functions, a set of enrichment analyses were conducted for the targets and the R packages “clusterProfiler,” “org.Hs.eg.db,” and “enrichplot” were used to analyze and visualize gene ontology (GO) and Kyoto Encyclopedia of Genes and Genomes (KEGG) pathways.

### 2.4 Molecular docking

To further validate the binding affinities between quercetin and proteins related to TC-diabetes, we performed molecular docking. The structure of quercetin was downloaded from PubChem (https://pubchem.ncbi.nlm.nih.gov/) website and modified in the ChembioOffice software. Receptor structures of proteins were obtained from the RCSB PDB database (https://www.rcsb.org/). Before docking, we used PyMOL (32, 33) and Autodock Tools (34) software to preprocess the receptors (proteins) and ligand (quercetin), including removing solvent and organics, adding hydrogen, calculating charge, and converting into pdbqt format. Finally, Autodock Vina was used to simulate the binding between the receptor and ligand by running the CMD command and PyMOL for visualization of the results, including potential binding sites and interaction forces.

### 2.5 Immunohistochemical staining

The expression of MMP3 protein in thyroid cancer tissues and normal thyroid tissues was obtained through the online website THE HUMAN PROTEIN ATLAS (http://www.proteinatlas.org/humanproteome).

### 2.6 Cell lines and cell culture

SW579 human thyroid cancer cells were purchased from the Cell Bank of the Chinese Academy of Sciences. The cells were cultured in RPMI-1640 medium supplemented with 10% fetal bovine serum (FBS) and 1% penicillin/streptomycin in a standard cell culture environment. To establish a high glucose environment, a high glucose RPMI-1640 medium containing 25 mmol/L glucose was used to culture SW579 cells. All cells were incubated in a cell culture incubator at 37°C with 5% CO2.

### 2.7 CCK8 test

SW579 cells were gathered from regular and high-glucose milieus and seeded into 96-well plates for a duration of 24 hours. Post adhesion to the plate walls, the cells were subjected to treatment by adding 400 μmol/L quercetin (with DMSO concentration below 0.1%) in the high-glucose condition. After a 24-hour incubation, 10 μl of CCK8 solution was dispensed into each well. Then, following a 1.5-hour incubation, the absorbance value at a wavelength of 450 nm was measured.

### 2.8 Real-time polymerase chain reaction

Using the Takara RR047A kit, 1 μg of total RNA underwent reverse transcription. For mRNA expression analysis, RT-PCR was carried out with the Takara RR420A TB Green^®^ Premix on an Applied Biosystems ViiA 7 system. Sample Ct values were evaluated via the 2^-△△^Ct approach, employing β-actin as the reference gene. Primer details are available in **Supplementary Table 1**.

### 2.9 Western blot analysis

10 μg of protein were fractionated via 10% SDS-PAGE and subsequently blotted onto a PVDF membrane. The membrane was blocked with 5% BSA and incubated overnight at 4°C with a primary antibody. After three 10-minute washes in TBST, it was incubated for 1 hour at room temperature with a secondary antibody. Protein bands were detected using an ECL reagent (Invitrogen). Antibodies used: anti-ß-actin (1:1000, 4970, Cell Signaling Technology, USA) and anti-MMP3 (1:2000, 17873-1-AP, Proteintech, China).

### 2.10 Statistical analysis

Statistical analyses utilized GraphPad Prism 10.0 and SPSS 20.0. Normally distributed data were presented as mean ± standard deviation (SD); non-normally distributed data as median and IQR. The Shapiro-Wilk test confirmed data normality. Group comparisons were done using the independent t-test or Mann-Whitney *U* test (as applicable). Multiple group comparisons employed one-way ANOVA (where suitable), with significance set at *p* < 0.05 (two-side).

## 3 Results

### 3.1 Common genes of TC-Diabetes and construction of prognostic model

**Figure 2:**
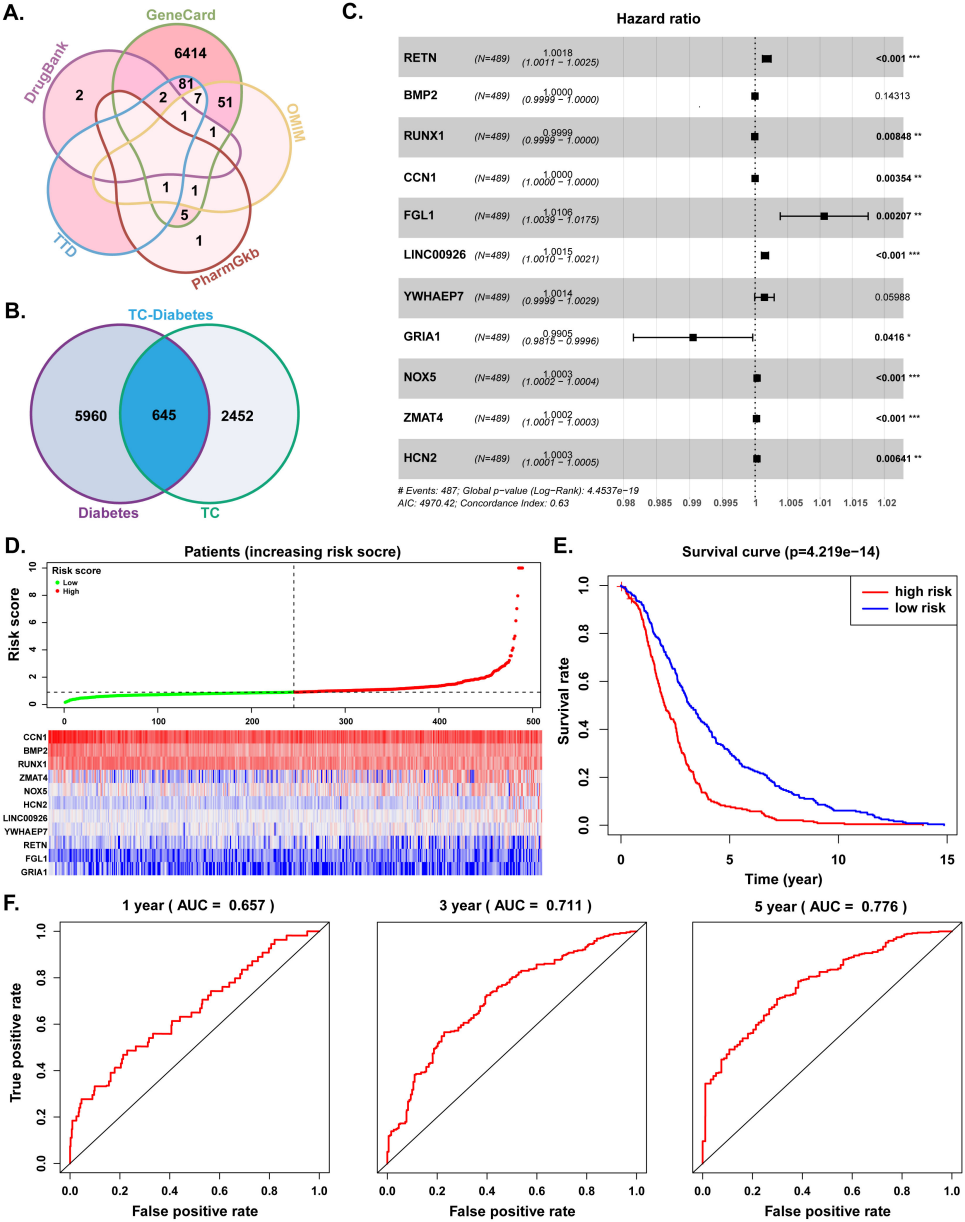
Screening TC-diabetes common genes and survival analyses. **(A)** Diabetes-related genes. **(B)** Common genes of diabetes and TC. **(C)** 11 Prognostic genes identified by multivariate Cox proportional hazards regression analysis. **(D)** Analysis of patients’ risk score using Cox proportional hazards regression (upper) and gene expression levels (bottom). **(E)** Survival curves of high-risk and low-risk groups. **(F)** 1-, 3- and 5-year ROC curve of risk score via multivariate Cox proportional hazards regression. AUC, area under the curve; TC, thyroid cancer.

**Figure 3:**
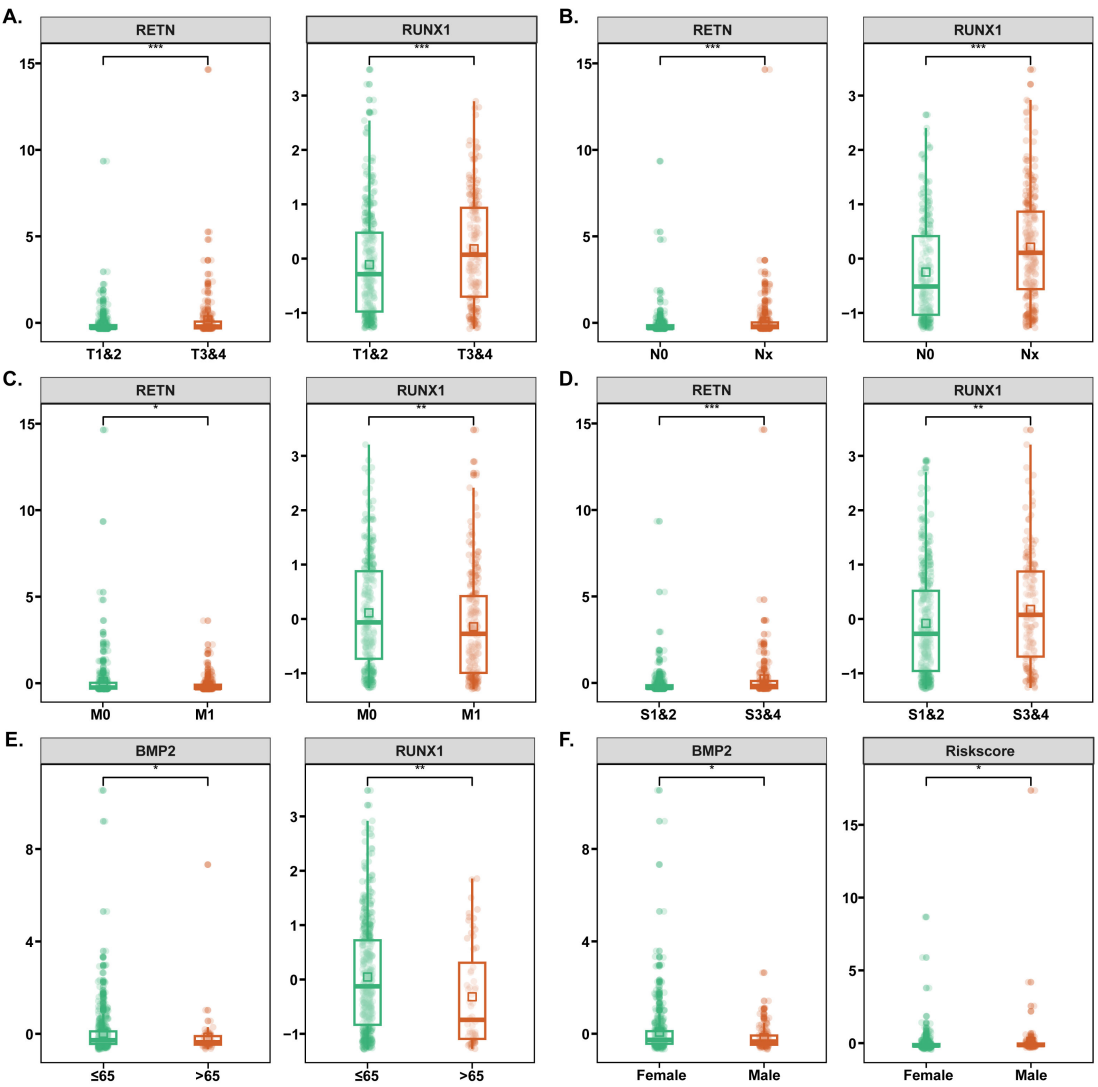
Clinical prognostic analyses of 11 genes. **(A–D)** Relationships between gene expressions and depth of tumor invasion, lymph node metastasis, distant metastasis of tumor, and TC stage, respectively. **(E)** Relationships between gene expressions and age of TC patients. **(F)** Relationships between gene expressions and gender in TC patients. TC, thyroid cancer. *p < 0.05, **p < 0.01, ***p < 0.001.

We searched for diabetes-related genes from GeneCard, OMIM, DrugBank, TTD, and PharmGKB databases, yielding a total of 6605 targets. Furthermore, by comparing gene expression matrix between the TC samples and normal samples from TCGA database, we obtained 3097 TC-related differential expressed genes (**Supplementary Figures S1A, B**). A total of 645 common genes from the intersection between diabetes- and TC- related genes were acquired for the following analyses (**Figures 2A, B**). Combining univariate Cox regression, Lasso regression, and multivariate Cox regression methods, we conducted a clinical prognostic risk model and identified 11 genes as prognostic targets, i.e., Resistin (RETN), Bone Morphogenetic Protein 2 (BMP2), Runt-Related Transcription Factor 1 (RUNX1), Cellular Communication Network Factor 1 (CCN1), Fibrinogen-Like 1 (FGL1), Long Intergenic Non-Protein Coding RNA 926 (LINC00926), Tyrosine 3-Monooxygenase/Tryptophan 5-Monooxygenase Activation Protein Epsilon Pseudogene 7 (YWHAEP7), Glutamate Ionotropic Receptor AMPA Type Subunit 1 (GRIA1), NADPH Oxidase 5 (NOX5), Zinc Finger Matrin-Type 4 (ZMAT4) and Hyperpolarization Activated Cyclic Nucleotide Gated Potassium and Sodium Channel 2 (HCN2) (**Figure 2C**, **Table 1**, **Supplementary Figures S1C, D**). Patients were then divided into high-risk and low-risk groups based on the risk scores, and the high-risk group was correlated with poorer survival outcomes relative to the low-risk group (**Figures 2D–F**). Clinical analysis indicated that RETN and RUNX1 expressions were associated with advanced TC stages, RETN, RUNX1 and ZMAT4 expressions were related to lymph node, and RUNX1, LINC00926 and GRIA1 expressions were linked to distant metastases (**Figures 3A–D**, **Supplementary Figures S2A–D**). Additionally, BMP2, RUNX1 and LINC00926 showed higher expression in younger patients, with BMP2 more highly expressed in female patients (**Figures 3E, F**, **Supplementary Figure S2E**).

**Table 1:** Multivariate Cox proportional hazards regression analysis.

Symbol	coef	HR	HR 95L	HR 95H	*P* value
RETN	0.00179	1.00179	1.00112	1.00246	0.00000
BMP2	-0.00003	0.99997	0.99993	1.00001	0.14313
RUNX1	-0.00006	0.99994	0.99990	0.99999	0.00848
CCN1	-0.00001	0.99999	0.99999	1.00000	0.00354
FGL1	0.01059	1.01065	1.00386	1.01748	0.00207
LINC00926	0.00151	1.00151	1.00096	1.00206	0.00000
YWHAEP7	0.00143	1.00143	0.99994	1.00292	0.05988
GRIA1	-0.00951	0.99053	0.98151	0.99964	0.04160
NOX5	0.00030	1.00030	1.00017	1.00043	0.00000
ZMAT4	0.00021	1.00021	1.00011	1.00031	0.00004
HCN2	0.00028	1.00028	1.00008	1.00048	0.00641

coef, Coefficient of multivariate cox proportional risk regression model; HR, hazard ratio; HR.95L, hazard ratio.95% low; HR.95H, hazard ratio.95% high.

### 3.2 Therapeutic targets of quercetin in THCA−diabetes and PPI network

**Figure 4:**
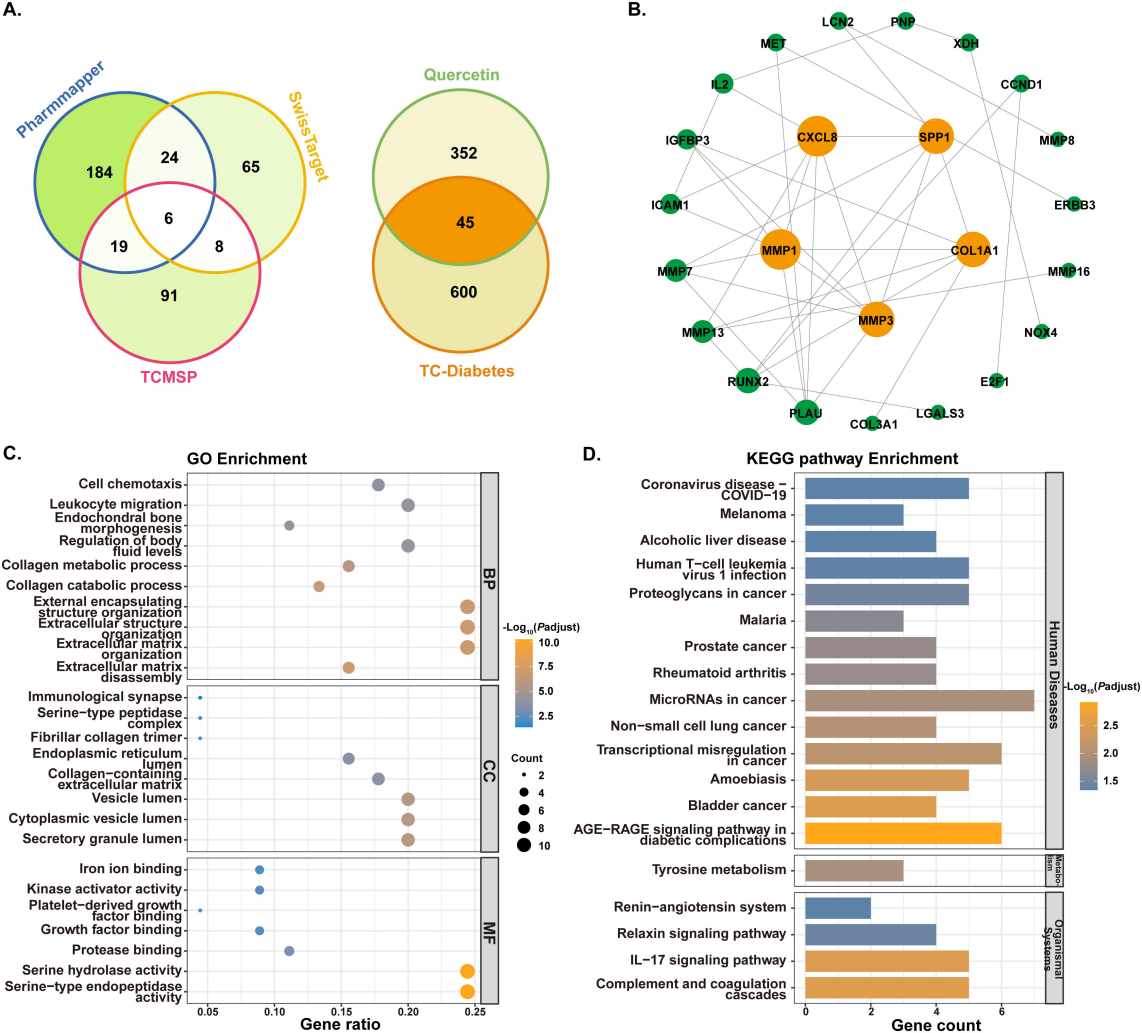
Screening targets of quercetin in treating TC-diabetes and functional enrichment analyses. **(A)** Venn diagrams of quercetin-related genes and common genes of quercetin genes and TC-diabetes genes. **(B)** Protein interaction network (PPI) of quercetin anti-TC-Diabetes genes. The circle nodes represent targets of quercetin in treating TC-diabetes with the oranges representing core targets screened by Cytoscape. **(C)** Gene ontology enrichment analysis. **(D)** Kyoto Encyclopedia of Genes and Genomes (KEGG) pathway enrichment analysis. TC, thyroid cancer.

After searching from the TCMSP database, PharmMapper database, and Swiss Target Prediction website and correcting gene deduplication through the UniProt database, a total of 397 quercetin-related genes were identified as targets (**Figure 4A**). Then, the intersection of these quercetin targets with the TC-diabetes targets yielded 45 common targets, suggesting that quercetin treats TC-diabetes via working on these key targets. To evaluate the potential therapeutic mechanism of quercetin on TC-diabetes, the 45 common genes were further included in a PPI network analysis using STRING. The PPI network was constructed and included 36 nodes and 45 edges as node sizes were proportional to the target degree. Most importantly, we identified a core PPI network based on the criteria of DC > 6 calculated by CytoNCA plug-in comprised of 5 core nodes and 25 edges (**Figure 4B**). The core gene targets with greater degree were Matrix Metalloproteinase 1 (MMP1), C-X-C Motif Chemokine Ligand 8 (CXCL8), Matrix Metalloproteinase 3 (MMP3), Secreted Phosphoprotein 1 (SPP1) and Collagen Type I Alpha 1 Chain (COL1A1).

### 3.3 Gene functional enrichment

To characterize the biological functions and pathways of the target genes related to quercetin in treating TC−diabetes, we performed functional enrichment analyses based on the 45 common genes. With regard to GO enrichment, a total of 144 biological features were confirmed including 129 biological processes (BP), 8 cellular components (CC), and 7 molecular functions (MF) (**Figure 4C**, **Supplementary File**). The enriched BP terms were involved extracellular matrix disassembly, extracellular matrix organization, extracellular structure organization, external encapsulating structure organization, collagen catabolic process, collagen metabolic process, regulation of body fluid levels, endochondral bone morphogenesis, leukocyte migration and cell chemotaxis; the enriched CC terms were involved secretory granule lumen, cytoplasmic vesicle lumen, vesicle lumen, collagen-containing extracellular matrix, endoplasmic reticulum lumen, fibrillar collagen trimer, serine-type peptidase complex and immunological synapse; the enriched MF terms were involved serine-type endopeptidase activity, serine hydrolase activity, protease binding, growth factor binding, platelet-derived growth factor binding, kinase activator activity and iron ion binding. In terms of KEGG pathways, a total of 19 pathways were identified including AGE−RAGE signaling pathway in diabetic complications, bladder cancer, amoebiasis, transcriptional misregulation in cancer, non−small cell lung cancer, microRNAs in cancer, rheumatoid arthritis, prostate cancer, malaria, proteoglycans in cancer, human T-cell leukemia virus 1 infection, alcoholic liver disease, melanoma, tyrosine metabolism, complement and coagulation cascades, IL-17 signaling pathway, relaxin signaling pathway and renin-angiotensin system (**Figure 4D**).

### 3.4 Molecular docking of the target genes

**Figure 5:**
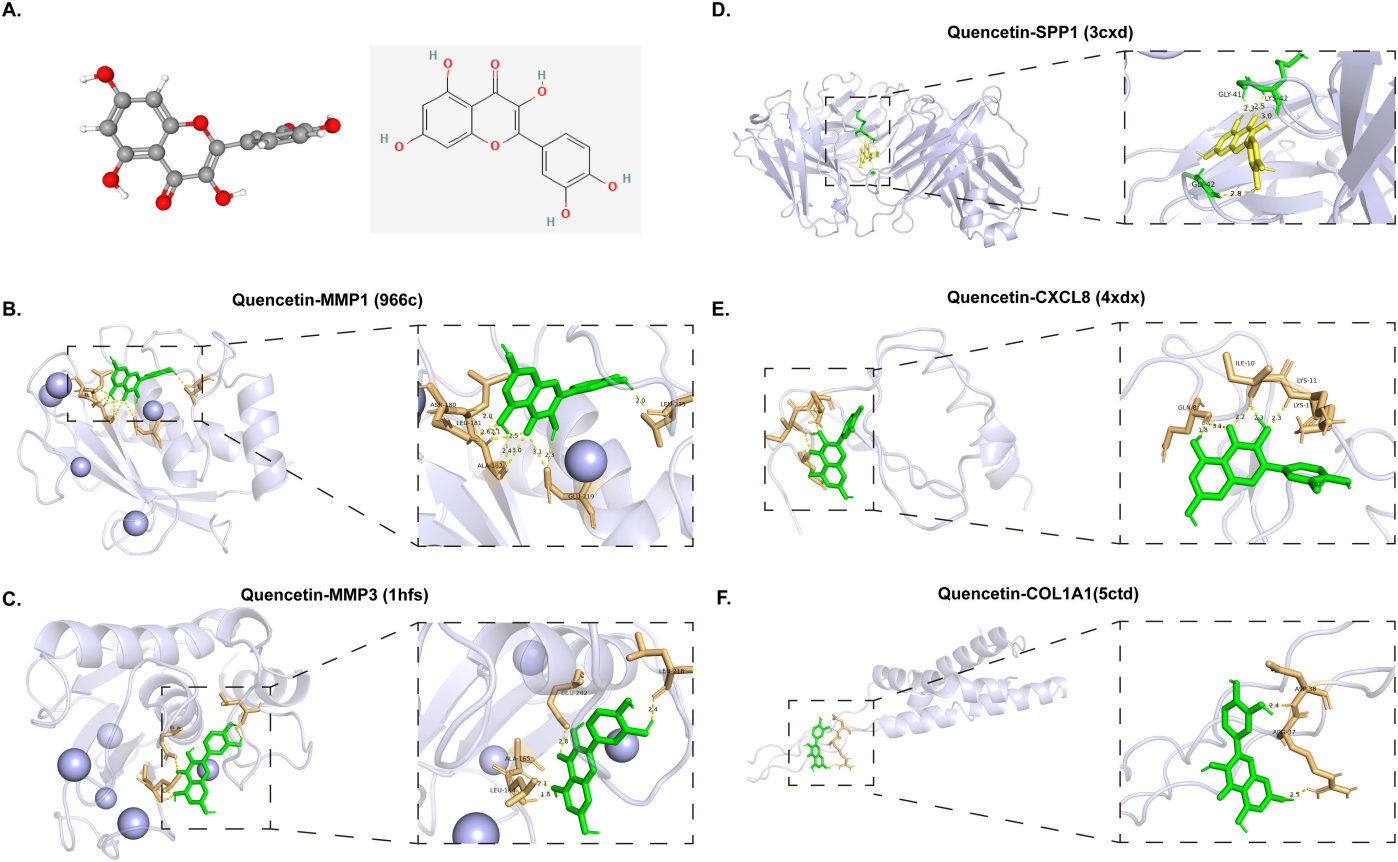
Molecular docking of core targets with quercetin. **(A)** Two and three-dimensional structure of quercetin. **(B–F)** Molecular docking of quercetin with MMP1 (966c), MMP3 (1hfs), SPP1 (3cxd), CXCL8 (4xdx) and COL1A1 (5ctd).

To validate the findings from network pharmacology, molecular docking was employed to estimate the affinity between quercetin and five core targets (MMP1, CXCL8, MMP3, SPP1 and COL1A1) involved in TC-diabetes treatment, separately. All core genes showed potential binding with quercetin (**Figure 5A**, **Table 2**). Quercetin’s binding interactions suggest it may alter the structure and function of these proteins, impacting downstream pathways to exert antitumor and antidiabetic effects. Specific hydrogen bond interactions were identified: MMP1: ASN-180, LEU-181, ALA-182, GLU-219 and LEU-235 (966c 1.90 Å); MMP3: ALA-165, LEU-164, LEU-218 and GLU-202 (1hfs 1.70 Å); SPP1: LYS-42, GLY-41, and GLY-42 (3cxd 2.80 Å); CXCL8: ILE-10, GLN-8 and LYS-11 (4xdx 0.95 Å); COL1A1: ASP-38 and ARG-37 (5ctd 1.60 Å) (**Figures 5B–F**). The docking results indicate that quercetin can form hydrogen bonds with these proteins, confirming potential interactions. Among the five proteins, quercetin showed the strongest binding affinity with MMP1 and MMP3, suggesting MMP1 and MMP3 are primary targets for quercetin in treating TC-diabetes.

**Table 2:** Binding site of quercetin to the protein expression product of quercetin anti-TC-diabetes core genes.

Component	PDB ID	△Gb (kcal/mol)	Bonds formed between functional groups of component and protein residues		
			Functional groups	Functional groups	Bond
Quercetin	966c	-10.2	O	ASN-180	H-bond
			O	LEU-181	H-bond
			O	ALA-182	H-bond
			O	GLU-219	H-bond
			O	LEU-235	H-bond
	1hfs	-10.0	O	ALA-165	H-bond
			O	LEU-164	H-bond
			O	LEU-218	H-bond
			O	GLU-202	H-bond
	3cxd	-7.9	O	LYS-42	H-bond
			O	GLY-41	H-bond
			O	GLY-42	H-bond
	4xdx	-7.6	O	ILE-10	H-bond
			O	GLN-8	H-bond
			O	LYS-11	H-bond
	5ctd	-5.2	O	ASP-38	H-bond
			O	ARG-37	H-bond

966c, MMP1; 1hfs, MMP3; 3cxd, SPP1; 4xdx, CXCL8; 5ctd, COL1A1; TC, thyroid cancer.

### 3.5 Quercetin inhibits the proliferation of human thyroid cancer cells and the expression of MMP3 in a high-glucose environment

**Figure 6:**
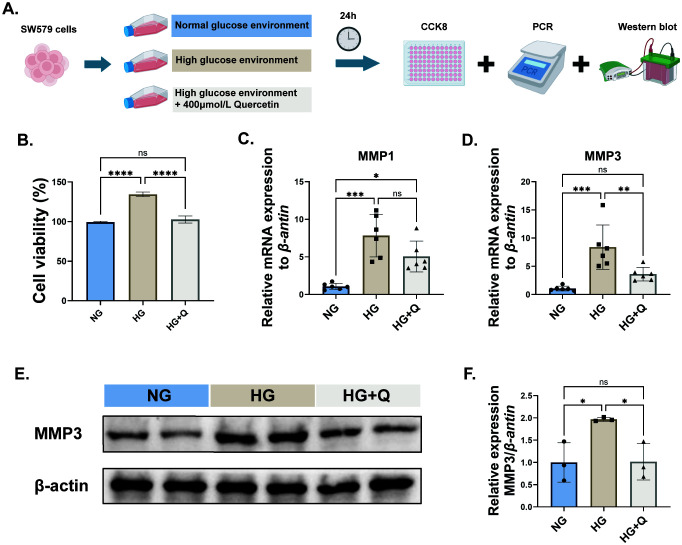
The effects of quercetin on human thyroid cancer (SW579) cells under high glucose environment. **(A)** Cell experiment design diagram; **(B)** CCK8 experiment to detect the cell viability of SW579 cells in each group; **(C)** PCR to detect the mRNA expression level of MMP1 in SW579 cells of each group; **(D)** PCR to detect the mRNA expression level of MMP3 in SW579 cells of each group; Western blot analysis of MMP3 protein expression in each group of cells **(E, F)**. NG, normal environment; HG, high glucose environment; HG+Q, high glucose environment with quercetin (400μmol/L). Normalization to Group NG. Data are presented as mean ± SD. *P < 0.05, **P < 0.01, ***P < 0.001.

To explore the effect of quercetin on the proliferation of thyroid cancer cells in a high-glucose environment, 400 μmol/L quercetin was applied to thyroid cancer cells in a high-glucose environment (**Figure 6A**). The experimental results showed that compared to the normal environment, the proliferation capacity of SW579 cells in the high glucose environment was significantly increased, while quercetin could offset this effect (*p* < 0.05, **Figure 6B**). Previous results showed that MMP1 and MMP3 might be potential targets of quercetin action. PCR detection results showed that quercetin could significantly inhibit the mRNA expression level of MMP3 in SW579 cells in a high glucose environment (*p* < 0.05, **Figure 6D**), but no significant difference was observed in MMP1 (**Figure 6C**). Further Western blot detection also showed that quercetin could significantly inhibit the expression level of MMP3 in SW579 cells in a high glucose environment (*p* < 0.05, **Figures 6E, F**). In addition, immunohistochemical results also indicated that the expression level of MMP3 protein in thyroid cancer tissues was significantly higher than that in normal thyroid tissues (**Supplementary Figure S3**).

## 4 Discussion

This is the first study investigating the molecular mechanisms underpinning quercetin in treating TC-diabetes. Both TC and diabetes belong to endocrine system diseases with various degrees of endocrine disorders. Although thyroid surgery, radioactive iodine therapy, and TSH suppression are effective measures in TC treatment, they can bring in a set of complications, in particular worsening in the condition of diabetes. Indeed, more secure and effective measures are of great necessity to control and treat TC-diabetes. Herb medicine from TCM plays an important role in the modern medical system as an adjuvant therapy, for its multiple efficacies in treating various complex diseases (9–12). Quercetin is among the widely prevalent flavonoid subclass of flavonols and abundantly in fruits and vegetables with a high medicinal value in clinic attributing to its remarkable anti-tumor and anti-diabetic effects (35–37). Although, previous studies have revealed the multi-target cancer inhibition and organ protection mechanism of quercetin from the perspective of thyroid cancer and diabetic nephropathy respectively, providing a theoretical basis for its synergistic application with standard therapy (38, 39), there is still a lack of research on the therapeutic effects of quercetin in thyroid cancer patients with comorbid diabetes. Consequently, this present study aims to explore the therapeutic effects and molecular mechanisms of quercetin on TC-diabetes by integrating bioinformatics, network pharmacology, and molecular docking, and further validating these observations with *in vitro* experiments.

In this study, a prognosis risk prediction model was built to evaluate the prognosis of the identified targets for TC-diabetes patients. Initially, we integrated and intersected the gene results by screening available databases. 645 common genes were identified between TC and diabetes, defined as TC-diabetes targets. Then, 11 targets (RETN, BMP2, RUNX1, CCN1, FGL1, LINC00926, YWHAEP7, GRIA1, NOX5, ZMAT4, and HCN2) were selected for subsequent prognostic analyses after conducting univariate Cox analysis, LASSO regression, and multivariate Cox regression analyses. Survival and ROC analyses showed that the performance of these targets in predicting survival was credible, regardless of short-term (1 year) or long-term (5 years) survival. Additionally, group comparison analyses between different conditions of clinical features revealed that these risk gene expression levels were related to ages, genders, and TNM stages of clinical patients. Taking together, DM exerts an adverse effect on the prognosis of TC and patients with such risk factors should be paid more attention to when considering their treatment and prognosis. In this study, we established a human thyroid cancer cell model under high glucose conditions. Our findings showed that the high glucose environment promotes cancer cell proliferation while quercetin can reverse the tumor-promoting effects of the high glucose environment. Therefore, it is of great significance to further study the therapeutic effect of quercetin on TC-diabetes and its potential mechanisms.

To investigate the molecular mechanisms of quercetin in treating TC-diabetes patients, we identified 45 common genes between quercetin targets and TC-diabetes targets by integrating data from three public pharmacology databases. Combining PPI network and CytoNCA plug-in in Cytoscape, five hub genes were further determined: MMP1, CXCL8, MMP3, SPP1 and COL1A. In addition, functional enrichment analyses of the 45 common genes indicated that they were related to different biological functions and pathways, which play an important role in cell migration and human diseases. Among them, extracellular matrix (ECM) structure organization is of great importance. Accumulated evidence clearly demonstrated over the last decade that ECM plays key roles in maintaining cell structure and function since it modulates cell signaling, properties and morphology (40). The mature ECM can also generate dynamic structural alterations in response to external stimuli (i.e., applied force or injury), which enables the tissue to maintain homeostasis and to respond to physiological challenges and stresses, including disease (41). With the advancement of molecular techniques, the role of ECM in tumor initiation, growth, and dissemination is getting more and more in-depth research that tumor ECM collaborates intricately with both malignant and stromal cells, initiating and facilitating the complex, multistage cascade that leads to metastatic capability (42). Notably, thyroid cancer-associated fibroblasts (CAFs) significantly contribute to the proliferation and advancement of thyroid cancer through the secretion of soluble factors and ECM proteins, which, in turn, profoundly influence the behavior and aggressiveness of thyroid cancer cells (43, 44). An animal experiments indicate that components of ECM could act as an essential mediator in the dynamic interplay between CAFs and tumor cells, ultimately promoting the growth of papillary thyroid tumors (45). Additionally, a recent review suggests that microgravity may become a novel therapy of TC by intervening cell growth processes such as apoptosis and structural changes of the cytoskeleton and the ECM (46). Therefore, the therapeutic perspectives targeting ECM of TC may be promising.

Molecular docking was carried out for the above five core genes (MMP1, CXCL8, MMP3, SPP1 and COL1A) to further estimate the binding affinities in receptor-ligand compounds and found that quercetin can produce hydrogen bonds with them. This suggested that these five hub genes may act as potential targets of quercetin in treating TC-diabetes. Among them, MMP1 and MMP3 genes had the strongest binding abilities with quercetin. Further *in vitro* experiments demonstrated that quercetin could inhibit the expression of MMP3 in human thyroid cancer cells under high glucose conditions. It is well acknowledged that matrix metalloproteinases (MMPs) are a family of proteolytic enzymes that are responsible for the degradation of ECM and basement membrane (47). They are crucially involved in a variety of physiological and pathological processes, such as tumor establishment, growth and metastasis. Emerging research has consistently demonstrated that MMP3 plays a pivotal role as a regulatory factor within a set of biological pathways to inhibit the tumor metastasis (48–50). Of most importance, Lin et al. found that through the suppression of MMP3, microRNA-17 could inhibit tumor cell migration and invasion both *in vitro* and *in vivo* experiments (51). Molecular research indicated that one of the transmembrane glycoproteins could induce MMPs including MMP3 and participate in carcinoma invasion in follicular thyroid carcinoma (52). Another pilot study indicated that the expression and secretion of MMP3 could be stimulated by members of the CC-chemokine family, and enhance thyroid cancer cell invasion and migration (53). MMP3 is also associated with anaplastic thyroid cancer (ATC) cell invasion and migration, suggesting it may be developed as a novel therapeutic target for ATC (54). Collectively, the expression of MMP3 gene was up-regulated in TC patients with the gene implicated in cell invasiveness and migration, such that it may serve as the therapeutic target of TC in future studies. Furthermore, considering that this study was not yet included in patient sample, the value of these molecular targets as clinical prognostic markers or predictors of treatment response will be further explored in the future.

## 5 Conclusion

In this study, we constructed a prognostic risk model for TC-diabetes via bioinformatics and evaluated the correlations between diabetes and TC prognosis under the conditions of clinical features. By integrating bioinformatics and network pharmacology, we further screened the targets of quercetin in treating TC-diabetes and analyzed its anti-tumor molecular mechanisms and biological functions. Molecular docking identified the drug binding affinities of targets with MMP3 exhibiting stronger affinities. *in vitro* experiments indicated that quercetin suppressed the growth and MMP3 expression of thyroid cancer cells under high glucose conditions, thereby alleviating the advancement of TC-diabetes. This provides a theoretical and practical foundation for treating TC-diabetes with quercetin.

## Data Availability

The original contributions presented in the study are included in the article/**Supplementary Material**. Further inquiries can be directed to the corresponding authors.

## References

[1] Boucai L Zafereo M Cabanillas ME. Thyroid cancer: A review. Jama. (2024) 331:425–35. doi: 10.1001/jama.2023.26348 38319329

[2] Chen DW Lang BHH McLeod DSA Newbold K Haymart MR. Thyroid cancer. Lancet. (2023) 401:1531–44. doi: 10.1016/S0140-6736(23)00020-X 37023783

[3] Diagnosis and classification of diabetes mellitus. Diabetes Care. (2013) 36 Suppl 1:S67–74. doi: 10.2337/dc13-S067 PMC3537273 23264425

[4] Dong WW Zhang DL Wang ZH Lv CZ Zhang P Zhang H. Different types of diabetes mellitus and risk of thyroid cancer: A meta-analysis of cohort studies. Front Endocrinol (Lausanne). (2022) 13:971213. doi: 10.3389/fendo.2022.971213 36213272 PMC9537385

[5] Schmid D Behrens G Jochem C Keimling M Leitzmann M. Physical activity, diabetes, and risk of thyroid cancer: a systematic review and meta-analysis. Eur J Epidemiol. (2013) 28:945–58. doi: 10.1007/s10654-013-9865-0 24243033

[6] Bezin J Gouverneur A Pénichon M Mathieu C Garrel R Hillaire-Buys D. GLP-1 receptor agonists and the risk of thyroid cancer. Diabetes Care. (2023) 46:384–90. doi: 10.2337/dc22-1148 36356111

[7] Wang M Gong WW Lu F Hu RY He QF Yu M. The association between diabetes and thyroid cancer risk: a hospital-based case-control study in China. BMC Endocr Disord. (2021) 21:21. doi: 10.1186/s12902-021-00684-y 33509182 PMC7845043

[8] Padovano M Scopetti M Tomassi R Manetti F D'Errico S Santurro A. Mapping complications in thyroid surgery: statistical data are useful for medico-legal management of a recurrent safety issue. Updates Surg. (2022) 74:1725–32. doi: 10.1007/s13304-022-01357-8 PMC9481495 36028654

[9] Jia Z Zhu X Zhou Y Wu J Cao M Hu C. Polypeptides from traditional Chinese medicine: Comprehensive review of perspective towards cancer management. Int J Biol Macromol. (2024) 260:129423. doi: 10.1016/j.ijbiomac.2024.129423 38232868

[10] Parekh HS Liu G Wei MQ. A new dawn for the use of traditional Chinese medicine in cancer therapy. Mol Cancer. (2009) 8:21. doi: 10.1186/1476-4598-8-21 19298677 PMC2664781

[11] Meng L Zhang C Yu P. Treating cancer through modulating exosomal protein loading and function: The prospects of natural products and traditional Chinese medicine. Pharmacol Res. (2024) 203:107179. doi: 10.1016/j.phrs.2024.107179 38615876

[12] Liao YH Lin CC Lai HC Chiang JH Lin JG Li TC. Adjunctive traditional Chinese medicine therapy improves survival of liver cancer patients. Liver Int. (2015) 35:2595–602. doi: 10.1111/liv.2015.35.issue-12 25875878

[13] Tang JL Liu BY Ma KW. Traditional chinese medicine. Lancet. (2008) 372:1938–40. doi: 10.1016/S0140-6736(08)61354-9 18930523

[14] Deepika Maurya PK. Health benefits of quercetin in age-related diseases. Molecules. (2022) 27:2498. doi: 10.3390/molecules27082498 35458696 PMC9032170

[15] Reyes-Farias M Carrasco-Pozo C. The anti-cancer effect of quercetin: molecular implications in cancer metabolism. Int J Mol Sci. (2019) 20:3177. doi: 10.3390/ijms20133177 31261749 PMC6651418

[16] Alizadeh SR Ebrahimzadeh MA. Quercetin derivatives: Drug design, development, and biological activities, a review. Eur J Med Chem. (2022) 229:114068. doi: 10.1016/j.ejmech.2021.114068 34971873

[17] Di Petrillo A Orru G Fais A Fantini MC. Quercetin and its derivates as antiviral potentials: A comprehensive review. Phytother Res. (2022) 36:266–78. doi: 10.1002/ptr.v36.1 PMC8662201 34709675

[18] Boretti A. Quercetin as a cancer chemopreventive or chemotherapeutic agent: Where we stand. Phytotherapy Res. (2022) 37:1227–31. doi: 10.1002/ptr.v37.4 36444390

[19] Shafabakhsh R Asemi Z. Quercetin: a natural compound for ovarian cancer treatment. J Ovarian Res. (2019) 12:55. doi: 10.1186/s13048-019-0530-4 31202269 PMC6570913

[20] Giuliani C Di Dalmazi G Bucci I Napolitano G. Quercetin and thyroid. Antioxidants (Basel). (2024) 13:1202. doi: 10.3390/antiox13101202 39456456 PMC11505551

[21] Dhanya R. Quercetin for managing type 2 diabetes and its complications, an insight into multitarget therapy. BioMed Pharmacother. (2022) 146:112560. doi: 10.1016/j.biopha.2021.112560 34953390

[22] Li D Jiang C Mei G Zhao Y Chen L Liu J. Quercetin alleviates ferroptosis of pancreatic β Cells in type 2 diabetes. Nutrients. (2020) 12:2954. doi: 10.3390/nu12102954 32992479 PMC7600916

[23] Mi S Zhu W Zhang X Wang Y Li T Wang X. Enhanced hypoglycemic bioactivity via RAS/raf-1/MEK/ERK signaling pathway by combining capsaicin and QUERCETIN from chili peppers. Mol Nutr Food Res. (2023) 67:e2200577. doi: 10.1002/mnfr.202200577 36907948

[24] Youl E Bardy G Magous R Cros G Sejalon F Virsolvy A. Quercetin potentiates insulin secretion and protects INS-1 pancreatic beta-cells against oxidative damage via the ERK1/2 pathway. Br J Pharmacol. (2010) 161:799–814. doi: 10.1111/j.1476-5381.2010.00910.x 20860660 PMC2992896

[25] Lin F Zhou W Yuan X Liu S He Z. Mechanistic study of quercetin in the treatment of hepatocellular carcinoma with diabetes via MEK/ERK pathway. Int Immunopharmacol. (2024) 142:113194. doi: 10.1016/j.intimp.2024.113194 39305892

[26] Wishart DS Feunang YD Guo AC Lo EJ Marcu A Grant JR. DrugBank 5.0: a major update to the DrugBank database for 2018. Nucleic Acids Res. (2018) 46:D1074–82. doi: 10.1093/nar/gkx1037 PMC5753335 29126136

[27] Liu X Ouyang S Yu B Liu Y Huang K Gong J. PharmMapper server: a web server for potential drug target identification using pharmacophore mapping approach. Nucleic Acids Res. (2010) 38:W609–14. doi: 10.1093/nar/gkq300 PMC2896160 20430828

[28] Zhou Y Zhang Y Lian X Li F Wang C Zhu F. Therapeutic target database update 2022: facilitating drug discovery with enriched comparative data of targeted agents. Nucleic Acids Res. (2022) 50:D1398–407. doi: 10.1093/nar/gkab953 PMC8728281 34718717

[29] Ru J Li P Wang J Zhou W Li B Huang C. TCMSP: a database of systems pharmacology for drug discovery from herbal medicines. J Cheminform. (2014) 6:13. doi: 10.1186/1758-2946-6-13 24735618 PMC4001360

[30] Gfeller D Grosdidier A Wirth M Daina A Michielin O Zoete V. SwissTargetPrediction: a web server for target prediction of bioactive small molecules. Nucleic Acids Res. (2014) 42:W32–8. doi: 10.1093/nar/gku293 PMC4086140 24792161

[31] Szklarczyk D Morris JH Cook H Kuhn M Wyder S Simonovic M. The STRING database in 2017: quality-controlled protein-protein association networks, made broadly accessible. Nucleic Acids Res. (2017) 45:D362–8. doi: 10.1093/nar/gkw937 PMC5210637 27924014

[32] Seeliger D de Groot BL. Ligand docking and binding site analysis with PyMOL and Autodock/Vina. J Comput Aided Mol Des. (2010) 24:417–22. doi: 10.1007/s10822-010-9352-6 PMC2881210 20401516

[33] Khanal P Patil VS Bhandare VV Patil PP Patil BM Dwivedi PSR. Systems and *in vitro* pharmacology profiling of diosgenin against breast cancer. Front Pharmacol. (2022) 13:1052849. doi: 10.3389/fphar.2022.1052849 36686654 PMC9846155

[34] Morris GM Huey R Lindstrom W Sanner MF Belew RK Goodsell DS. AutoDock4 and AutoDockTools4: Automated docking with selective receptor flexibility. J Comput Chem. (2009) 30:2785–91. doi: 10.1002/jcc.v30:16 PMC2760638 19399780

[35] Tang SM Deng XT Zhou J Li QP Ge XX Miao L. Pharmacological basis and new insights of quercetin action in respect to its anti-cancer effects. BioMed Pharmacother. (2020) 121:109604. doi: 10.1016/j.biopha.2019.109604 31733570

[36] Zang X Cheng M Zhang X Chen X. Quercetin nanoformulations: a promising strategy for tumor therapy. Food Funct. (2021) 12:6664–81. doi: 10.1039/D1FO00851J 34152346

[37] Biswas P Dey D Biswas PK Rahaman TI Saha S Parvez A. A comprehensive analysis and anti-cancer activities of quercetin in ROS-mediated cancer and cancer stem cells. Int J Mol Sci. (2022) 23:11746. doi: 10.3390/ijms231911746 36233051 PMC9569933

[38] Sun Y Xie W Kang N Yi J Ruan X Hu L. To explore the inhibitory mechanism of quercetin in thyroid papillary carcinoma through network pharmacology and experiments. Dis Markers. (2022) 2022:9541080. doi: 10.1155/2022/9541080 36510497 PMC9741536

[39] Ma X Hao C Yu M Zhang Z Huang J Yang W. Investigating the Molecular Mechanism of Quercetin Protecting against Podocyte Injury to Attenuate Diabetic Nephropathy through Network Pharmacology, MicroarrayData Analysis, and Molecular Docking. Evid Based Complement Alternat Med. (2022) 2022:7291434. doi: 10.1155/2022/7291434 35615688 PMC9126727

[40] Karamanos NK Theocharis AD Piperigkou Z Manou D Passi A Skandalis SS. A guide to the composition and functions of the extracellular matrix. FEBS J. (2021) 288:6850–912. doi: 10.1111/febs.v288.24 33605520

[41] Mouw JK Ou G Weaver VM. Extracellular matrix assembly: a multiscale deconstruction. Nat Rev Mol Cell Biol. (2014) 15:771–85. doi: 10.1038/nrm3902 PMC4682873 25370693

[42] Karamanos NK Piperigkou Z Passi A Götte M Rousselle P Vlodavsky I. Extracellular matrix-based cancer targeting. Trends Mol Med. (2021) 27:1000–13. doi: 10.1016/j.molmed.2021.07.009 34389240

[43] Avagliano A Fiume G Bellevicine C Troncone G Venuta A Acampora V. Thyroid cancer and fibroblasts. Cancers (Basel). (2022) 14:4172. doi: 10.3390/cancers14174172 36077709 PMC9455043

[44] Shin E Koo JS. Cell component and function of tumor microenvironment in thyroid cancer. Int J Mol Sci. (2022) 23:12578. doi: 10.3390/ijms232012578 36293435 PMC9604510

[45] Jin X Deng Q Ye S Liu S Fu Y Liu Y. Cancer-associated fibroblast-derived periostin promotes papillary thyroid tumor growth through integrin-FAK-STAT3 signaling. Theranostics. (2024) 14:3014–28. doi: 10.7150/thno.94207 PMC11103496 38773979

[46] Krüger M Melnik D Kopp S Buken C Sahana J Bauer J. Fighting thyroid cancer with microgravity research. Int J Mol Sci. (2019) 20:2553. doi: 10.3390/ijms20102553 31137658 PMC6566201

[47] Kraiem Z Korem S. Matrix metalloproteinases and the thyroid. Thyroid. (2000) 10:1061–9. doi: 10.1089/thy.2000.10.1061 11201850

[48] Masuda T Fukuda A Yamakawa G Omatsu M Namikawa M Sono M. Pancreatic RECK inactivation promotes cancer formation, epithelial-mesenchymal transition, and metastasis. J Clin Invest. (2023) 133:e161847. doi: 10.1172/JCI161847 37712427 PMC10503799

[49] Li N Xu X Liu D Gao J Gao Y Wu X. The delta subunit of the GABA(A) receptor is necessary for the GPT2-promoted breast cancer metastasis. Theranostics. (2023) 13:1355–69. doi: 10.7150/thno.80544 PMC10008743 36923530

[50] Seehawer M Li Z Nishida J Foidart P Reiter AH Rojas-Jimenez E. Loss of Kmt2c or Kmt2d drives brain metastasis via KDM6A-dependent upregulation of MMP3. Nat Cell Biol. (2024) 26:1165–75. doi: 10.1038/s41556-024-01446-3 PMC11251985 38926506

[51] Lin YH Liao CJ Huang YH Wu MH Chi HC Wu SM. Thyroid hormone receptor represses miR-17 expression to enhance tumor metastasis in human hepatoma cells. Oncogene. (2013) 32:4509–18. doi: 10.1038/onc.2013.309 23912452

[52] Omi Y Shibata N Okamoto T Obara T Kobayashi M. The role of CD147 in the invasiveness of follicular thyroid carcinoma cells. Thyroid. (2012) 22:383–94. doi: 10.1089/thy.2010.0426 22280229

[53] Zeng W Chang H Ma M Li Y. CCL20/CCR6 promotes the invasion and migration of thyroid cancer cells via NF-kappa B signaling-induced MMP-3 production. Exp Mol Pathol. (2014) 97:184–90. doi: 10.1016/j.yexmp.2014.06.012 24984269

[54] Ma Y Cang S Li G Su Y Zhang H Wang L. Integrated analysis of transcriptome data revealed MMP3 and MMP13 as critical genes in anaplastic thyroid cancer progression. J Cell Physiol. (2019) 234:22260–71. doi: 10.1002/jcp.v234.12 31081124

