# Supplementary material for: Mechanistic study of quercetin in the treatment of thyroid cancer with diabetes based on network pharmacology and in vitro experiments
Source: Front Endocrinol (Lausanne). 2025 Jun 12;16:1537799. doi: 10.3389/fendo.2025.1537799 (PMC12197918; doi:10.3389/fendo.2025.1537799)
Supplement: Supplementary file 1 [file DataSheet1.doc]

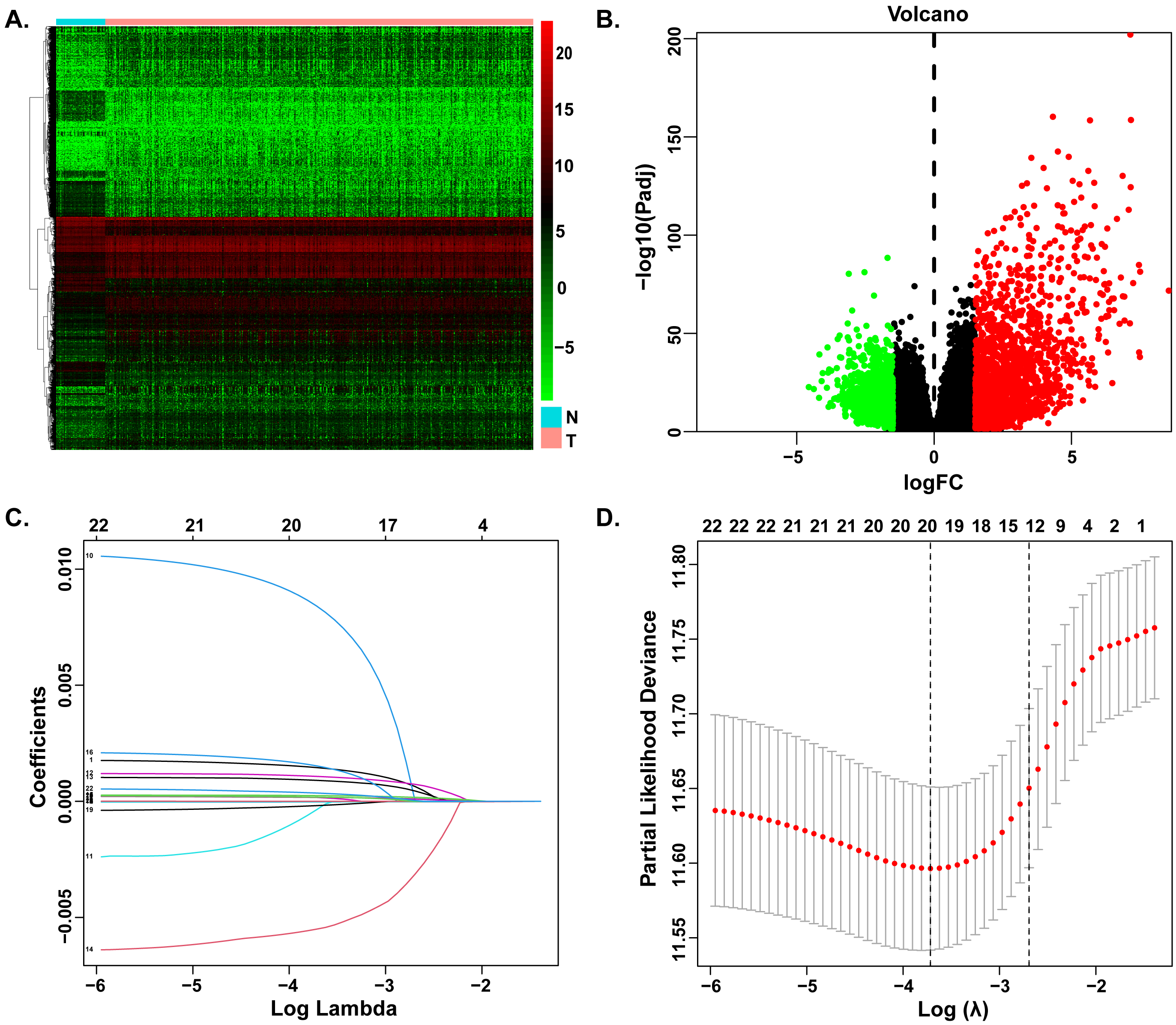


**Figure S1** Screening genes. A. Heatmap of differentially expressed genes in the analysis of microarray data. Red represents significantly up-regulated genes; Green represents significantly down-regulated genes. B. A volcano map of differentially expressed genes between tumor tissue samples and normal tissue samples in the TCGA-THCA dataset. The X coordinate is log_2_ (fold change) and the Y coordinate is -log_10_ (*P*_adj_). Each dot represents a gene. Green dots are significantly down-regulated genes. Red dots represent significantly up-regulated genes. Black dots indicate unaltered expressed genes. C. The variation characteristics of the coefficient of variables; D. the selection process of the optimum value of the parameter λ in the Lasso regression model by cross-validation method.


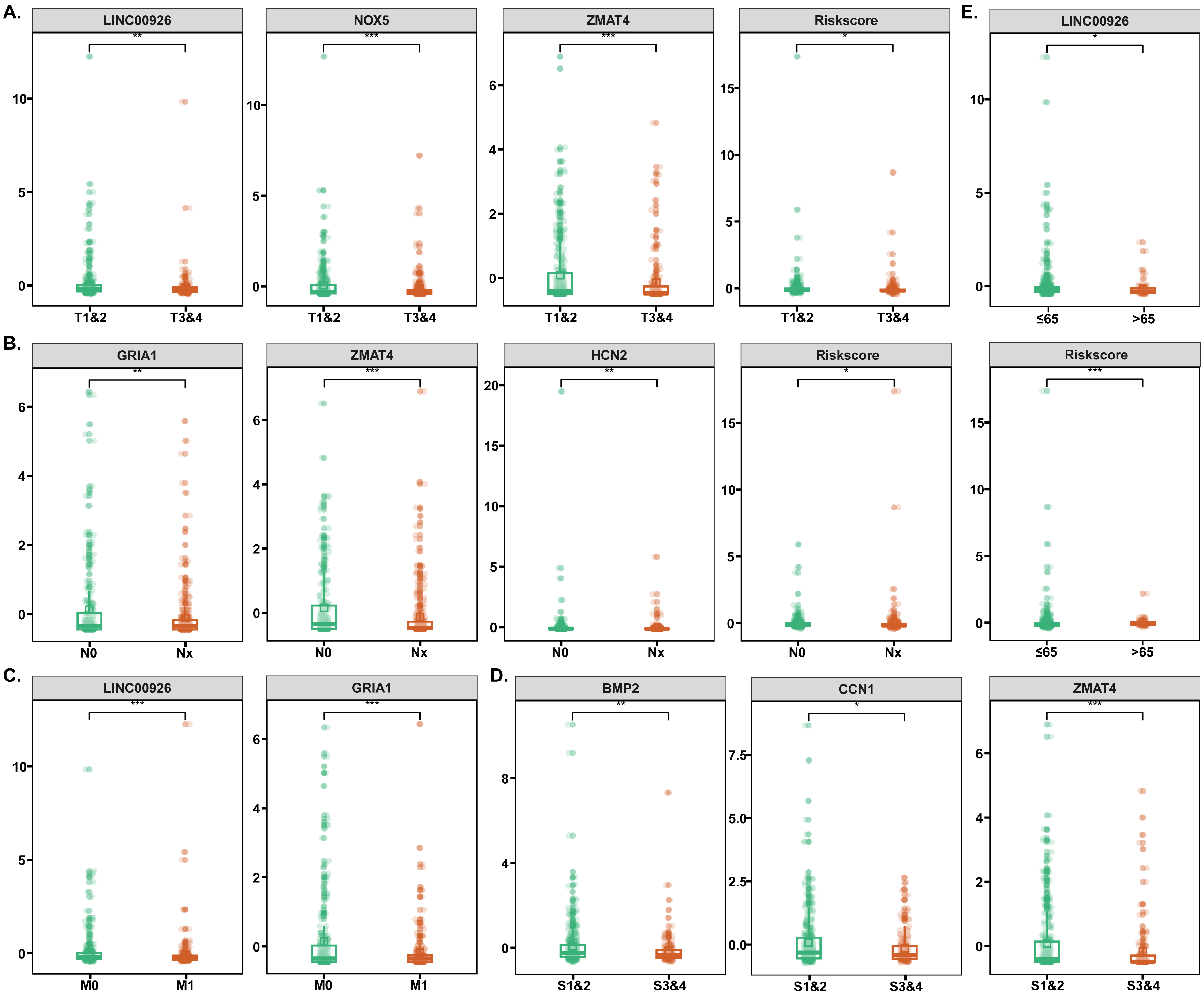


**Figure S2** Clinical prognostic analyses of 11 genes. A-D. Relationships between gene expressions and depth of tumor invasion, lymph node metastasis, distant metastasis of tumor and TC stage, respectively. E. Relationships between gene expressions and age of TC patients. Abbreviations: TC, thyroid cancer.


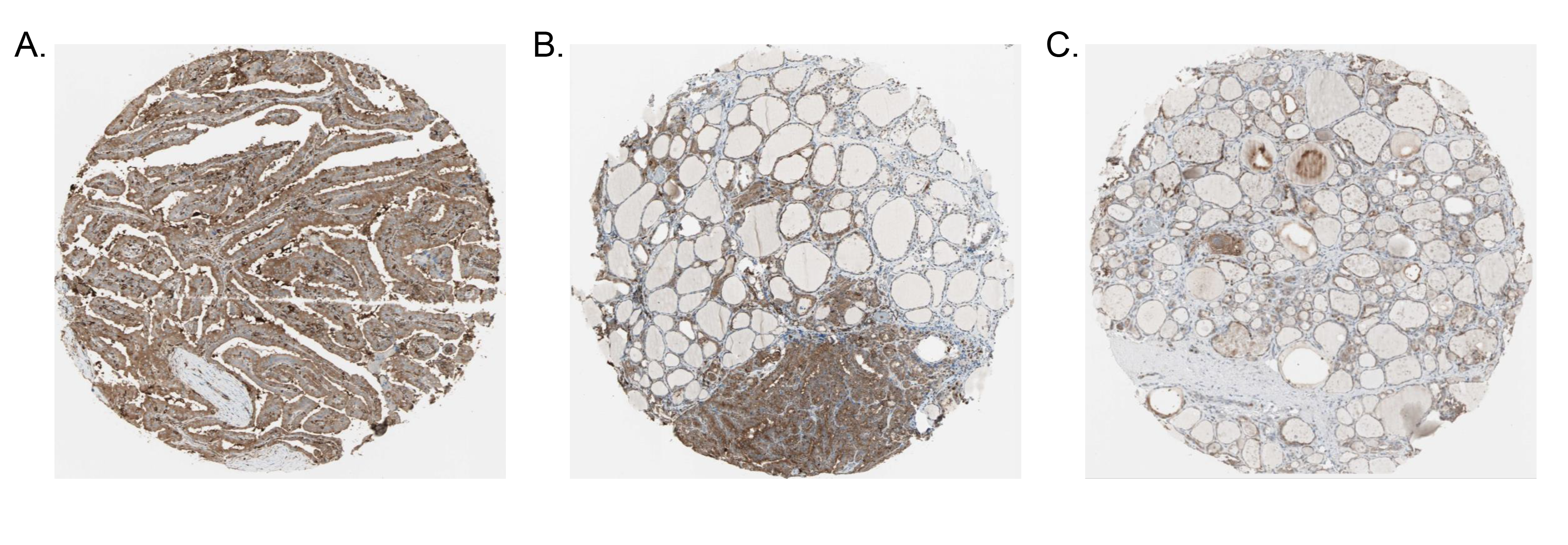


**Figure S3** Immunohistochemical analysis of MMP3 expression in thyroid cancer tissues (A-B) and normal thyroid tissue (C).

**Supplementary Table 1**. Primer sequences

| Gene | Species | Primer | Sequence (5′–3′) |
| --- | --- | --- | --- |
| MMP1 | Human | Forward primer | AAAATTACACGCCAGATTTGCC |
|  |  | Reverse primer | GGTGTGACATTACTCCAGAGTTG |
| MMP3 | Human | Forward primer | AGTCTTCCAATCCTACTGTTGCT |
|  |  | Reverse primer | TCCCCGTCACCTCCAATCC |
| β-actin | Homo sapiens | Forward primer | TCATGAAGTGTGACGTGGACATC |
|  |  | Reverse primer | CAGGAGGAGCAATGATCTTGATCT |
